# Ibrutinib-Induced Paronychia and Periungual Pyogenic Granuloma

**DOI:** 10.7759/cureus.32943

**Published:** 2022-12-25

**Authors:** Awadh Alamri, Yara Alghamdi, Atheer Alghamdi, Dhai B Albogami, Omar Shahada, Aseel AlHarbi

**Affiliations:** 1 Dermatology, King Abdulaziz Medical City, Jeddah, SAU; 2 General Practice, King Abdullah International Medical Research Center, Ministry of the National Guard - Health Affairs, Jeddah, SAU; 3 College of Medicine, King Saud Bin Abdulaziz University for Health Sciences College of Medicine, Jeddah, SAU; 4 College of Medicine, King Saud bin Abdulaziz University for Health Sciences, King Abdullah International Medical Research Center, Jeddah, SAU; 5 Dermatology, King Salman bin Abdulaziz Medical City, Medina, SAU; 6 Dermatology, College of Medicine and Surgery, Taibah University, Medina, SAU

**Keywords:** bruton tyrosine kinase inhibitor, tyrosine kinase receptor inhibitors, nail diseases, beau's line, pyogenic granuloma

## Abstract

Nail changes elicited by Ibrutinib are relatively infrequent but are reported in the literature. Herein, we report on two cases that developed Ibrutinib-induced nail toxicities. A 63-year-old female, with relapsing mantle cell lymphoma on Ibrutinib 560mg/day for seven months developed paronychia, onychomadesis, Beau’s lines, nail fragility, and brittleness over fingernails and toenails. On the other hand, an 80-year-old male with chronic lymphoid leukemia developed a bloody papule with hemorrhagic crust and nail-plate abnormalities. Skin toxicities manifested eight months after initiating Ibrutinib therapy. From a clinical perspective, Ibrutinib-induced chronic paronychia and PG have been established. All other PG triggers have been ruled out. After the cessation of Ibrutinib, the PG improved for both cases. The exact pathogenesis of PG induced by Ibrutinib is not yet understood but it had been compared to retinoid-related changes. Thus, further research and reporting of similar cases should be done to further understand the pathophysiology of such manifestations.

## Introduction

Pyogenic granuloma (PG), also known as lobular capillary hemangioma, is a benign vascular tumor. It manifests as a solitary and rapidly growing papule or nodule. The exact pathophysiology is still debated. Nonetheless, it is elicited by a variety of triggers like trauma, pregnancy, infection, immune deficiency, certain medications, or imbalance in the body’s stimulatory and inhibitory markers [1]. The commonly implicated medications in PG are cetuximab (epidermal growth factor inhibitor), imatinib (tyrosine kinase inhibitor), tacrolimus (calcineurin inhibitor), or HIV protease inhibitors [1-3]. One of the less reported medications that induce PG is Ibrutinib (tyrosine kinase inhibitor) [4-6]. Herein, we describe two patients who developed Ibrutinib-induced PG and nail-plate abnormalities.

## Case presentation

Case 1

A 63-year-old woman with relapsing mantle-cell lymphoma (MCL) was referred with nail changes and bloody papules that were not improving on topical fusidic-acid 2%. The dermatological changes manifested four months after receiving oral Ibrutinib 560mg/day. There were no other medications or other MCL-directed therapy.

**Figure 1:**
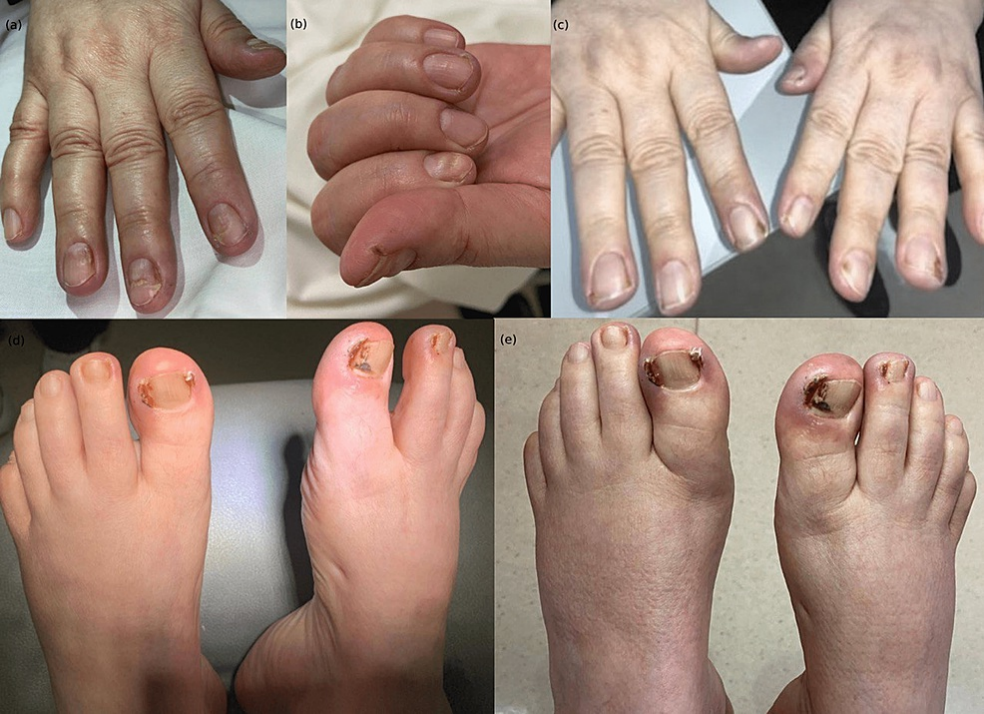
Ibrutinib-induced paronychia and PG in a 63-year-old with MCL. (a) Fragile and brittle nails with trachyonychia, linear splitting, horizontal splitting, and shedding of proximal nails (onychomadesis) of the fingernails of the right hand. A crusted papule is evident over the index finger. (b) Erythematous papule covered by hemorrhagic crust over the thumb. (c) A distant view of the hands showing periungual PG over fingernails. (d) Periungual PG of toenails. Bloody papule covered by hemorrhagic crust over both big toenails and second toenails of right feet. (e) Ibrutinib-induced periungual PG over toenails in a 63-year-old female with mantle cell lymphoma.

A dermatological examination revealed hemorrhagic crusted papules, onychomadesis, beau’s lines, and multiple periungual raised bright erythematous lesions over fingernails and toenails (Figures 1a-1e). There was no history of onychocriptosis or trauma preceding the findings or any new medication intake except the Ibrutinib 560mg daily that was introduced seven months ago. The first impression was chronic paronychia associated with PG and managed with topical mupirocin 2% Potassium Permanganate 0.1% solution, and oral Amoxicillin/Clavulanic acid 625mg three times daily for one week.

A follow-up examination after one month showed no improvement with the prescribed regimen. Thus, silver nitrate was done for the right big toe and liquid-nitrogen cryotherapy was applied for the right second toe. Based on clinical evaluation, a diagnosis of Ibrutinib-induced paronychia and periungual PG was established.

Follow-up after three weeks showed mild improvement over fingernails. Two electrocautery sessions done for big toenails showed mild improvement. The discontinuation of Ibrutinib was deferred and the dose was minimized to 420mg/day and showed moderate improvement, but PG persisted. At the follow-up examination for three months, Ibrutinib was eventually stopped, and PG has resolved with no relapse.

Case 2 

**Figure 2:**
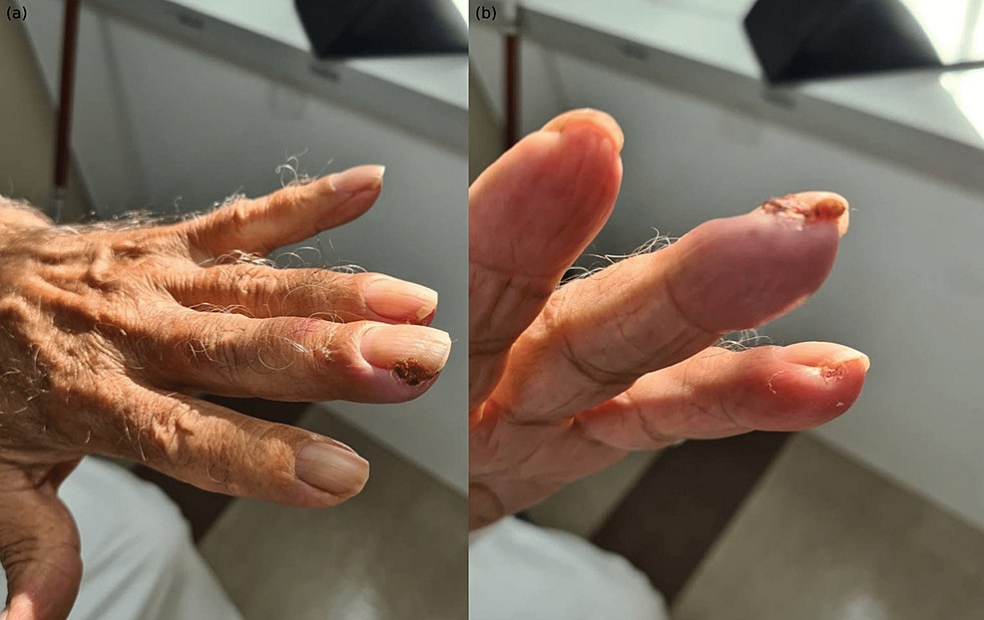
Ibrutinib-induced periungual PG in an 80-year-old male with CLL. (a) Bloody papule with hemorrhagic crust over the third digit of the left hand. Brittle nails with longitudinal ridges. (b) Pedunculated erythematous papule with hemorrhagic crust over the fourth digit of the left hand. Dermatitis and desquamation over the tip of the fifth digit.

An 80-year-old male with chronic lymphoid leukemia (CLL) developed skin changes with bleeding lesions and was referred by the treating oncologist for dermatological evaluation. The scaly bleeding papule with hemorrhagic crust started eight months after initiating Ibrutinib 420mg daily. There were no CLL-targeted therapies, or any other medications introduced. No history of trauma or similar lesions before. A dermatological examination yielded two erythematous papules with hemorrhagic crust (Figures 2a, 2b). A clinical diagnosis of Ibrutinib-induced PG was established. Symptomatic management with fusidic-acid and hydrocortisone cream (Fucidin-H) was given when needed. At the follow-up examination for two months, Ibrutinib was stopped by the treating oncologist and PG has subsided with no relapse.

## Discussion

Ibrutinib is an oral Bruton’s tyrosine kinase-inhibitor (BTKi) that is approved for the treatment of patients with CLL after one prior therapy, MCL, small lymphocytic lymphoma (SLL), Waldenstrom’s macroglobulinemia, and chronic graft-versus-host disease after failure of at least one systemic therapy [3]; and by the European Medicines Agency for patients with CLL, and adult patients with relapsed or refractory MCL [2,3].

Frequent side effects range from diarrhea and fatigue to serious implications to the heart like atrial fibrillation and bleeding events leading to drug discontinuation in 2%-26% of patients [2,3]. Both cases delineated in this report had Ibrutinib stopped which resulted in PG and nail-plate changes subsiding with no relapse.

Ibrutinib-induced nail-plate changes have been explored before by Bitar et al. [7]. Those changes have been examined in 66 participants with long-term use of Ibrutinib and reported that 67% have experienced fingernail brittleness and 22.7% experienced toenail brittleness. Nail changes manifested over the course of six to nine months. The temporal association of Ibrutinib-induced nail changes in this report is similar to reports from Bitar et al. [7]; as Case 1 presented with nail-plate abnormalities after four months, while Case 2 manifested nail changes after eight months. Bitar et al. [7] have only explored nail-plate abnormalities but did not report any periungual PG manifesting in any participant.

Ibrutinib-induced periungual PG has been reported before in case and nail-plate changes have been addressed in three case reports (Table 1) [4-6]. Management differed among reported cases from liquid nitrogen surgical resection, boric acid soak, and topical corticosteroids (Table 1) [4-6].

**Table 1:** Ibrutinib-induced nail changes that have been reported in literature.

	Age (years)	Gender	Clinical diagnosis	Co-morbidities	Medication	Anatomical involvement	Presentation	Temporal association	Management
Ibrutinib-induced nail-plate abnormalities									
Manica, et al. [4]	79	Female	Chronic lymphocytic leukemia	-	Ibrutinib oral daily dose of 420 mg	Toenails	Longitudinal ridges, horizontal splitting, and distal shedding. Dermatitis and superficial desquamation. Fragile and brittle nails. Trachyonychia with linear splitting. Talon noir.	6 months	Improvement with cryotherapy and biotin.
	53	Male		-		Thumb, fingernails, and toenails		4 months	Biotin 2.5 mg
Yorulmaz, et al. [5]	40	Female		Acromegaly, type 2 diabetes mellitus, and hypo-thyroidism.	Ibrutinib oral daily	Toenails	Erythematous, swelling, and serosanguinous discharge on its lateral nail fold. Paronychia.	2 months	Boric acid soak and topical corticosteroids. Patient lost follow up.
Ibrutinib-induced PG and nail-plate abnormalities									
Roussel, et al. [6]	69	Male	Chronic lymphocytic leukemia	-	Ibrutinib oral daily	Toenail	Brittle nails. Serosanguinous discharge on its lateral nail fold. Ungual PG over third toenails.	4 months	Complete resection was performed and resulted in no relapse.
	52	Male		-		Toenail	Fingertip and ungual PG of the right hallux.	11 months	Complete resection, but PG relapsed after 9 months.
	82	Female	Mantle cell lymphoma	Atrial fibrillation secondary to Ibrutinib	Ibrutinib oral daily initiated then tapered to 280 mg	Toenails	Brittle nails and PG on the intergluteal fold of the right hallux nail.	24 months	Cryotherapy resulted in a partial response and tapering the dose resulted in no relapse.

Ibrutinib reported adverse events like atrial fibrillation and bleeding have resulted in drug discontinuation; there were no reports of skin toxicities that led to discontinuation. In this report, Case 1 has mild improvement after reducing Ibrutinib dose from 560mg-daily to 420mg-daily. Complete resolution was achieved after Ibrutinib discontinuation in both reported cases.

The exact mechanism of Ibrutinib inducing nail changes remains unclear. Yorulmaz et al. [5] suggested that Ibrutinib-induced nail changes are similar to retinoid-related manifestations. Ibrutinib causes nail brittleness by disturbing the disulfide bonds between cysteine residues and diminishing the covalent binding of the cysteine residue at the active site of BTK on the B-cell resulting in brittle and fragile nails [5].

Baran [8] discussed retinoid-related side effects that developed due to desquamative dermatitis. Desquamative particles act as a foreign body in the lateral nail groove, leading to inflammation and granulation tissue formation. Thus, retinoid-related abnormalities can cause paronychia, excess granulation tissue, and PG formation [8].

## Conclusions

This paper illustrated the presentation of two cases that developed Ibrutinib-induced PG and nail changes. Ibrutinib-induced PG and nail abnormalities are rarely reported and such cases are vital for treating oncologists and dermatologists to recognize. As these Ibrutinib-induced skin toxicities could lead to drug discontinuation due to intolerable PG and nail-plate changes; also, as its effect on the quality of life. The PG secondary to Ibrutinib could be managed by many topical preparations, but physical therapy with resection, cryotherapy, and electrocautery showed mild to moderate improvement. In this report, Ibrutinib discontinuation resolved PG and nail-plate abnormalities in both cases.
